# Progress in Medicine

**Published:** 1899-12-02

**Authors:** 


### Progress in Medicine.

### RHEUMATISM AND GOUT.

**Nodules.**—G. F. Still and F. Poynton[1] regard these structures as identical with those found on the endocardium. They appear to consist of three zones, the central one is homogeneous, staining as fibrinous exudations do, the next is cellular, and the outer one fibrous. They probably commence as an exudation, and appear and vanish in some cases within a few days. The question has been raised whether the nodules in erythema nodosum are of the same origin. Washbourne and others, however, consider the rheumatic theory of this disease at least unproven.

**Bacteriology.**—A. S. Wohlman[2] describes the various organisms which have been put forward as peculiar to acute rheumatism, and shows that Achalme's bacillus has been accepted by many other authors, while its special properties help to explain the common clinical symptoms of the disease. Thus it is killed by minute quantities of salicylic acid, gives off a sour smell by the evolution of lactic and other acids, and when grown in sterilised urine causes a copious precipitation of urates. When injected into small animals it causes local œdema and death, and its presence in the blood often leads to an invasion by various forms of cocci, by which it seems to be replaced in the later stages. In appearance it sometimes resembles the anthrax bacillus, stains by Gram's method or feebly alkaline methylene blue, and forms spores on the third or fourth day. It is found in the blood and grows best in horse bouillon mixed with a little glycerine.

**Hyperpyrexia.**—H. G. Langwell[3] discusses at length this complication. He finds that it occurs in about ·5 per cent. of adult cases, very rarely in children, and not often in females. It is found most frequently in the second week of the disease, and preceded by delirium. If the temperature is not affected by full doses of salicylates, and especially if the pains and sweating cease, in such cases hyperpyrexia is to be feared. He does not believe it depends on visceral complications, and insists on the importance of using cold applications or baths instead of antipyretic drugs at an early stage, if either the pyrexia is persistent or delirium appears without complications to account for it.

**Phlebitis** is rarely seen, but two cases are recorded by Garnier.[4] One occurred in a late stage of a fourth attack after endocarditis, where the femoral and other veins were attacked, followed by similar trouble in the arm. The other was found in the first week of illness in a patient who had also gone through several attacks. The affection is most often found in the lower limbs. Pericarditis is said by F. J. Poynton[5] to be secondary to or concurrent with myocarditis. Extreme dilatation of the heart is very often present, and the changes in the myocardium are not limited to the surface, but extend through its substance.

**Treatment.**—Mosher[6] commences with large doses of salicylates, and afterwards keeps up the action with salophen. For chronic cases Falk employs piperazin, which controls the pain as well as improving the general state. Tongalin is praised by C. W. Canan in teaspoonful doses every hour in hot water, while Lee Beck finds general Faradisation of use in subacute cases. Chauvet thinks the galvanic bath of value in chronic rheumatism, and G. Singer employs intravenous injections of corrosive sublimate for acute attacks with any pyæmic symptoms, or where a single joint is affected or salicylates are counter-indicated. From a sixth to a third of a grain are injected daily or every two days. For obstinate sciatica the hydrochloric acid treatment is sometimes useful. In 16 cases R. A. Bayliss recently reported that two were quite cured and 11 relieved. The strong B.P acid is painted on the skin by a glass brush in fine lines two or three inches long over the tender spots. When dry the limb is wrapped in cotton wool and bandaged loosely. It may be repeated every night if no redness or irritation of the skin is produced. For neuritis and neuralgia Shirley Jones,[7] at Droitwich, gives hot brine baths with rest to the limb. When the pain is severe hot brine compresses are used, and as the acute stages pass away a douche over the nerve is employed of a temperature varied according to the case. Professor Lemoine[8] has had remarkable success with methylene blue. For seven cases of sciatica he gave as much as 50 centigrammes (7½ grains) daily. In 80 per cent. of the cases the pain was removed. In obstinate cases of migraine, trigeurinal neuralgia, zona, the abdominal crises of tabes, some cases of angina pectoris, diabetes, and rheumatism, even of the gonorrheal form, methylene blue has been found to give relief. Several authors have successfully employed it for cases of malaria which failed to recover under quinine. It is best given in pills with half its weight of powdered nutmeg. The dose is one to six grains three times daily, but the patient should be warned not to be alarmed at the blue colour of his urine. Wohlemuth[9] suggests aspirin or acetyl salicylic acid as preferable to salicylate of soda from its unirritating character. He advises that it should be given in powder in doses of three grammes daily, and records some successful cases of rheumatism and kindred disorders treated by it. Altdorfer,[10] in recommending Turkish baths for various rheumatic disorders, lays stress on the increased leucocytosis, excretion of $CO_2$, and alkalinity of the blood brought about by their use. The cold douche is important except where the heart is weak, and for therapeutic purposes he advises two baths daily, which he considers have no exhausting effect. In the early stage of phthisis and in melancholia a course of baths are often useful, but in the latter the cold douche should not be applied to the head. Lewis Jones[11] reviews the various contrivances for using radiant heat and light as therapeutic agents. In the Winternitz cabinet the patient's body is exposed to several incandescent lamps, where, it is said, the heat comes from a source at very high temperature, and is radiated rather than conducted. It penetrates the tissues more deeply, has a more stimulating effect, and produces sweating more rapidly. The dowsing apparatus is of a similar type, while Margaret Cleaves reports good results from the use of arc lights. However, L. Jones does not think it proved that radiant heat has any therapeutic properties beyond those of ordinary forms, though Hedley claims some advantages for it. Bowles remarks that all heat is radiant, even after conduction, and the value of the light rays is unknown. Though many observers testify to the good results of hot air treatment, cases are given by Hyde and others where crippled joints have afterwards become more and more fixed, and seemed to be actually injured, by too frequent and prolonged use of this method.

[1] Brit. Med. Jour., April. 8. [2] Brit. Med. Jour., Nov. 11. [3] New York Med. Jour., Mar. 4. [4] Lancet, April 22. [5] Brit. Med. Jour., May 13. [6] Clinic Jour., Mar. 22. [7] Brit. Med. Jour., June 17. [6] Treatment, p. 145, 1899. [9] Brit. Med. Jour., July 1. [10] Dublin Jour. Med. Sci., Aug. 1. [11] Brit. Med. Jour., Mar. 18.

### DISEASES OF THE DIGESTIVE ORGANS.

(*Concluded from page* 130.)

**Colitis.**—This seems to occur usually in large institutions, and very rarely in private families. A severe outbreak took place in the Derby Asylum, and out of 54 cases 23 persons died.[53] There were patches of congestion going on to destruction of the mucous membrane over large areas, and where the small intestine was attacked sharply cut ulcers were also found. Large doses of ipecacuanha seemed to be the only effective remedy. The bacillus enteritidis sporogenes was isolated, an organism which varies enormously in virulence, and which resists high degrees of heat when present in cooked food. It was found in an epidemic of diarrhœa at St. Bartholomew's Hospital last year, which was traced to rice pudding, and in the present instance non-virulent forms were discoved in the sweepings from the wards of the asylum. Beardoff[54] shows that many cases of intestinal dyspepsia are due to chronic ulcers of the colon and rectum. He employs injections of normal saline solution, and of weak carbolic and listerin. Many cases of chronic diarrhœa with symptoms of intestinal absorption, especially if a tender area over the colon can be made out, are relieved by similar treatment. T. Macpherson[55] describes a case of simple ulcerative colitis, where diarrhœa, emaciation, and pain in the left side of the abdomen and tenderness were the chief symptoms. Vomiting and an aphthous state of the mouth, as well as considerable pyrexia, were also present. The disease ran its course to a fatal termination, unchecked by various remedies. Masses of clear cut ulcers were afterwards found in the colon with much pigmentation of the rest of the mucous membrane. He notices that the characteristic griping pains with bloody offensive motions usually seen were here absent. We may notice that in membranous colitis injections of a very dilute solution—five grains to a quart of picric acid —have been recommended after previous lavage.[56] Four cases of supposed volvulus of the transverse colon are reported by G. H. Hunter,[57] which were treated by rotating the body round its long axis while in the horizontal position. In all of them the pain was most severe in the right lumbar region, about two inches below the tip of the gall bladder, and in two cases a swelling could be detected. As turning over towards the right side increased the pain, the writer considered the volvulus was twisted from right to left. To unwrap this twist, the patient was directed to turn three times over to the left while lying straight. Each time the pain was lessened, and finally complete recovery was obtained, with disappearance of the swelling where it existed.

**Congenital Dilatation.** — T. P. Crozier Griffiths[58] analyses some 40 cases of this affection, which presented the common symptoms of constipation and distension. Sometimes purgatives were successful, and many patients had attacks of pain and vomiting. Any part of the large intestine may be enlarged; the colon is generally thickened, and often ulcerated. Massage, electricity, and enemata may be tried. Osler thinks that laparotomy should be done early.

**Peritonitis.**—It would seem that the gonococcus may be the cause of acute peritonitis. Cushing,[59] of Johns Hopkins Hospital, refers to four cases. In one of them the peritoneum was found covered with yellow fibrin, and coverslips tested during the operation showed the gonococcus, which had gained entrance through the Fallopian tubes. The diffuse general inflammation thus caused by the gonococcus is apparently rare, but its existence is without doubt. Flexner[60] divides forms of peritonitis into (*a*) primary, which occurs independently of any of the organs enclosed, or of trauma; (*b*) secondary, from wounds and diseases of the intestinal tract, the last being the most common. In primary forms the infection usually enters through the lymphor blood. The bacteria found here are the pus cocci, B. coli, pyocyaneus, proteus, &c. An instance of perforation in typhoid which was closed by adherent omentum is recorded by D. J. M. Miller.[61] The discovery of this condition was made after death from a second perforation. A similar case is recorded by Buhl. An important point is the recognition of a pre-perforative stage. A localised peritonitis occurs when the ulcer nearly reaches the surface, and Miller and Cushing think it is shown by the gradual onset of continuous pain complained of by the patient himself, with tenderness and slight rigidity. Filatoff[62] lays down that chronic serous peritonitis other than tubercular does occur sometimes. The patient does not lose flesh or become so pale as in the tubercular form. The abdomen is not tense or painful. There are no nodules, and the fluid is free to seek its level. The temperature soon becomes normal, and the prognosis is good. Rest in bed, with light food, and attention to any diarrhœa present, is usually all that is required. Hahn[63] describes a very rare disease, which, however, is found not infrequently in pigs—pneumatosis cystoides. Soft tumours were felt in the abdomen, and these turned out to be clusters of small cysts situated on the intestines. They were easily broken, and contained gas. In this patient, a drover, some were excised, and a gradual recovery took place.

Another rare disease, an infection by pentastoma constrictum, is described by A. J. Chalmers[64] as occurring in a negro patient at Accra. He was emaciated, and suffered from a severe cough and pulmonary congestion, with an enlarged liver and spleen. Post-mortem, the parasites were found moving about in the abdominal cavity and attached to various organs. Both in the lungs and liver there were many cysts containing living animals, and in the intestines also numerous parasites were seen. Nothing is known of the life history of the creature, and the diagnosis would be very difficult unless it were found in the fæces or sputa.

53 Laneet, Sept. 23. 54 Internat. Med. Mag, May. 55 Australas. Med. Gaz., April. 56 New York Med. Jour., April 15. 57 Lancet, Aug. 13. 58 Amer. Jour. of Med. Sci., Sept. 59 Columbus Med. Jour., May. 60 Internat. Med. Mag., p. 297. 61 Lancet, June 24. 62 Med. Rec., April 22. 63 Brit. Med. Jour., July 1. 64 Lancet, June 24.

